# Improving return-to-work after childbirth: design of the Mom@Work study, a randomised controlled trial and cohort study

**DOI:** 10.1186/1471-2458-7-43

**Published:** 2007-03-29

**Authors:** Suzanne GM Stomp-van den Berg, Mireille NM van Poppel, Ingrid JM Hendriksen, David J Bruinvels, Kimi Uegaki, Martine C de Bruijne, Willem van Mechelen

**Affiliations:** 1 Body@Work, Research Centre Physical Activity, Work and Health, TNO-VUmc, VU University Medical Centre, Van der Boechorststraat 7, 1081 BT Amsterdam, The Netherlands; 2 Department of Public and Occupational Health, EMGO Institute, VU University Medical Centre, Van der Boechorststraat 7, 1081 BT Amsterdam, The Netherlands; 3 TNO Quality of Life, Wassenaarseweg 56, 2333 AL Leiden, The Netherlands; 4 Research Centre for Insurance Medicine: collaboration between AMC-UWV-VUmc, P.O. Box 7057, 1007 MB Amsterdam, The Netherlands; 5 Unit for Health Technology Assessment, EMGO Institute, VU University Medical Centre, Van der Boechorststraat 7, 1081 BT Amsterdam, The Netherlands

## Abstract

**Background:**

Many women suffer from health problems after giving birth, which can lead to sick leave. About 30% of Dutch workers are on sick leave after maternity leave. Structural contact of supervisors with employees on maternity leave, supported by early medical advice of occupational physicians, may increase the chances of return-to-work after maternity leave. In addition, to understand the process of sick leave and return-to-work after childbirth it is important to gain insight into which factors hinder return-to-work after childbirth, as well, as which prognostic factors lead to the development of postpartum health complaints. In this paper, the design of the Mom@Work study is described.

**Methods:**

The Mom@Work study is simultaneously a randomised controlled trial and a cohort study. Pregnant women working for at least 12 hours a week at one of the 15 participating companies are eligible to participate. The supervisors of these pregnant employees are randomised at 35 weeks pregnancy into the intervention group or control group. During maternity leave, supervisors in the intervention group contact their employee six weeks after delivery using a structured interview. When employees do not expect to return to their jobs at the end of their scheduled maternity leave due to health problems, the supervisor offers early support of the occupational physician. Supervisors in the control group have no structural contact with their employees during maternity leave. Measurements take place at 30 weeks pregnancy and at 6, 12, 24 and 52 weeks postpartum. In addition, cost data are collected. For the RCT, primary outcome measures are sick leave and return-to-work, and secondary outcome measures are costs, health, satisfaction with intervention and global feelings of recovery. Outcome measures for the cohort are pregnancy-related pelvic girdle pain, fatigue and depression. Finally, a number of prognostic factors for return-to-work and for the development of complaints will be measured.

**Discussion:**

The Mom@Work study will provide important information about return-to-work of employees after giving birth. Results will give insight in prognosis of postpartum sick leave and complaints. Also, the role of supervisors and occupational physicians in successful return-to-work after childbirth will be clarified.

## Background

Many women experience health problems during the first year after childbirth [1-4]. Common postpartum problems are fatigue, bowel problems, lack of sleep, postpartum depression, urinary incontinence, back pain and pelvic pain [1,4-6]. These common physical and mental health problems can lead to sick leave and long-term sickness absence from work [1].

In The Netherlands, every pregnant employee has the right to receive paid maternity leave, also called pregnancy and delivery leave. The total duration of this leave is 16 weeks, divided over four to six weeks before the delivery term and 10–12 weeks thereafter. About 90% of Dutch working women had the intention to return-to-work (RTW) after the birth of their first child [7]. A Dutch study described that 29% of working women were absent due to sickness for two weeks or more after maternity leave [1]. Sick leave postpartum was often long-term; in 55% of cases the sickness absence exceeded 12 weeks. The most occurring causes of sick leave postpartum were: pelvic pain (30%), back pain (26%), fatigue (23%), and mental problems (14%). [1]. Despite an abundance of literature about regular RTW and sick leave, little is known about the process of RTW after childbirth. A broad range of factors may contribute to RTW or sick leave, for instance factors like breastfeeding, childcare, social support, work load, distress, health, lack of sleep or health complaints. Therefore, it is important to gain insight into which factors hinder RTW after the end of maternity leave, as well as into the prognostic factors which lead to the development of these complaints.

There are several possible explanations for this high sick leave and postponed RTW postpartum. The postpartum care for women is probably to fragmented. At six weeks postpartum, women have their last contact with the midwife or obstetrician, who have limited time and who give only little or no information about RTW. At Youth Health Care centres only the child's health is considered and due to limited time the mother's health is often neglected. If there are health problems, women consult their general practitioner just before the scheduled maternity leave ends. In addition, these women with postpartum health complaints may not receive timely medical attention of their occupational physician (OP), who is currently not involved until 12–18 weeks postpartum. This may be too late; health complaints may have existed for a considerable time, while early intervention after the onset of complaints seems to be important [8-11], because prognosis for RTW becomes worse due to long-term sick leave [12]. An accompanying problem is the lack of communication between these different medical health care providers. Furthermore, women are not supported in the way they should be by their OP after maternity leave[13]. Postpartum, women receive less support than in 'normal' situations, meaning that those women are less activated to RTW. Finally, the role of supervisors as case-managers for this group of employees is less well defined, especially during maternity leave.

Supervisors often serve as a case-manager in case of sick leave in The Netherlands, however, they do not play a similarly important role in supporting the employee and recognizing problems for RTW that occur during maternity leave. Most companies in The Netherlands do not have a special policy for structured contact during maternity leave. Nieuwenhuijsen et al. recommended that supervisors should communicate frequently with employees during (prolonged) sick leave and should hold follow up meetings often in order to facilitate RTW in general [14]. It seems plausible that the supervisor can play a key role in preventing work disability by acting as a case-manager and stimulating earlier contact and involvement of the OP during maternity leave.

A structural contact between supervisors and women on maternity leave, supported by early medical advice of occupational physicians during maternity leave may increase the chances of RTW after maternity leave or reduce the incidence of sick leave after maternity leave. This paper describes the design of the Mom@Work study, a randomised controlled trial (RCT) and cohort study, conducted at Body@Work, Research Centre Physical Activity, Work and Health, TNO-VUmc in Amsterdam, The Netherlands. The Mom@Work study has three aims. The first aim is to investigate the effectiveness and cost-effectiveness of an early intervention in reducing sick leave in women after maternity leave in a randomised clinical trial. Our hypothesis is that supervisors who have structural contact with their employees during maternity leave combined with occupational health care, when needed, will be more effective in reducing the number of sick leave days after maternity leave than supervisors who do not. The second aim of the study is to examine which factors contribute to RTW for women after childbirth. The third aim is to examine which factors contribute to the development of the following postpartum complaints: pregnancy-related pelvic girdle pain, depression and fatigue. The second and third aim are examined in the cohort study.

## Methods/design

### Study design

The study is designed as a simultaneous randomised controlled trial (RCT), to assess effectiveness and cost-effectiveness of the intervention in reducing postpartum sick leave, and a cohort study, to assess incidence and determinants who contribute to postpartum RTW and to the development of postpartum health complaints. The Medical Ethics Committee of VU University Medical Centre at Amsterdam, The Netherlands approved the study design, study protocol, and informed consent procedure. All participants must provide a written informed consent.

### Recruitment of companies

For practical reasons, large companies with a predominantly female workforce were preferred to enter the study. It has been estimated that annually about five percent of the women becomes pregnant in The Netherlands. Van Beukering (2002) found that health care companies had higher sick leave rates before and after maternity leave than other companies. Therefore, health care companies were over sampled in the study.

Ninety-three companies were approached and ultimately 15 companies consented to participate in the study. The participating companies included university hospitals (n = 3), child care companies (n = 3), regional hospitals (n = 2), a health care group including a regional hospital, elderly care and home care (n = 1), a ministry of the Dutch government (n = 1), a chain of supermarkets (n = 1), a youth health care company (n = 1), an occupational health service provider (n = 1), a chain of special travel shops (n = 1) and a chain of pharmacists (n = 1). The company size ranged between 391 and 52,481 employees.

### Recruitment of study population

All pregnant women who were working between January 1, 2004 and March 31, 2006 at one of the 15 participating companies in The Netherlands were able to participate in the Mom@Work study. When a woman submitted the request for maternity leave, a human resources (HR) staff member of the participating company sent a short information letter about the study, a study leaflet, two response cards and a postal envelope to this woman. If the woman was interested in participating, she was asked to complete the 'Yes' response card and mail it to the research staff. The 'Yes' response card contained screening questions for both the RCT and cohort.

#### Inclusion and exclusion criteria cohort study

Pregnant women were eligible to enter the cohort study when they met the following inclusion criteria: age between 18 and 45 years; employment for a minimum of 12 hours a week before maternity leave and intention to RTW after maternity leave. Furthermore, they had to give written informed consent and be sufficiently fluent in the Dutch language. Women were not admitted to the RCT or cohort study if they met any of the following exclusion criteria: definitely not returning to work after maternity leave; miscarriage or delivery before 27 weeks; receiving a full disability benefit; or submitting an application for a full disability benefit.

#### Specific inclusion and exclusion criteria RCT

The inclusion criteria for the RCT were stricter than for the cohort study. Women could only enter the RCT if they met the criteria of the cohort study and the following inclusion criteria: employment for a minimum of 12 hours a week until six months after the delivery and working for the same employer before and after maternity leave.

**Figure 1:**
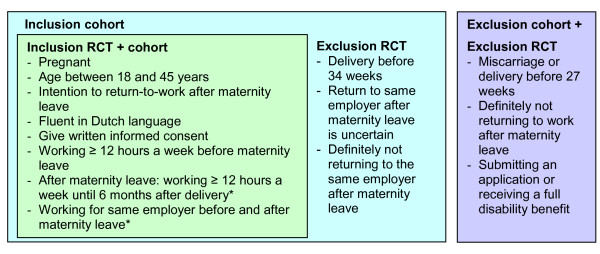
**Inclusion and exclusion criteria RCT and cohort study**. * Specific inclusion criteria RCT.

The specific exclusion criteria for the RCT were: a delivery before thirty-four weeks; return to the same employer after maternity leave is uncertain; and definitely not returning to the same employer after maternity leave. All participants of the RCT took also part in the cohort study. Women, who were not admitted to the RCT, could enter the cohort study if they met the inclusion criteria of the cohort study. Figure 1 shows an overview of the inclusion and exclusion criteria for the RCT and cohort study.

### Group allocation

Due to the fact that most supervisors managed a team of employees, the possibility existed that more than one employee would participate in the Mom@Work study. Thus, randomisation took place at the level of the supervisor rather than the participating woman. For each participating company, a randomisation list was computer-generated by an independent statistician. Because it was not known how many employees of each company would be participating in the study and some companies had a small number of employees, random allocation in blocks of four was chosen. Each block contained two intervention and two control group allocations in a random sequence. The details of the randomisation were printed on tickets and sealed into envelopes. The outside of the envelope was marked with the company's number and a number indicating the rang order. Supervisors were randomised when the participant was 35 weeks pregnant. When the researcher was informed in time that the participant gave birth before 34 weeks, the participant was not randomised, but still included in the cohort study.

Each week during the enrolment period, a list of supervisors who were to be randomised, was printed. The researcher allocated the supervisors of the women to the intervention group or to the control group by opening the next numbered envelope. The research assistant was responsible for sending emails and information to the supervisors who were randomised into the intervention group. The supervisors who were randomised into the control group did not receive any information and were not informed that their employee was participating in the study.

### Treatment groups for the RCT

#### Intervention group

The intervention concerns a minimal intervention strategy. The participant receives a phone call from her supervisor six weeks after childbirth and, if needed, support of the occupational health service providers (OHSP). The aim of the intervention is to prevent sick leave by only one phone call and by instigating earlier help by OHSP for the health problems. When health problems are pointed out during the phone call from the supervisor, the women can receive support of the OHSP at least five to seven weeks earlier than usual.

During the structured telephone contact the supervisor uses a standardised interview in the form of a checklist, and asks questions about the women's well being, the child's well being, child care, breast feeding, anticipated work problems, and the expectation of returning to work after maternity leave. When a woman expects that she is not returning to work after maternity leave, or has doubts about it, she is offered support of the OHSP by the supervisor. Only when she wants this support, the OHSP is called in. If requested by the participant, another representative of the company such as another supervisor, executive, line-manager, head of department or HR-advisor initiates this contact.

When a woman does not anticipate any problem with RTW, the supervisor does not offer further support, and he or she only asks when the woman will start working again. Women who do not expect any problem and who do not receive any early help of the OHSP, but who ultimately delay returning to work after maternity leave due to illness, receive the usual sick leave care.

If indicated during the phone call, the supervisor, participant or research team calls the OHSP that the woman needs their support on behalf of the study. A regular, but accelerated procedure for sick leave is initiated by the OHSP. In most cases, an appointment for early medical advice of the occupational physician with the participant is made within one week, instead of the usual six. The OP follows an OHSP complaint specific protocol, if available.

The supervisors receive a thorough instruction to carry out the structural contact. First, they receive an email to check if they are actually the supervisor of the participant and to check their personal data like work address and phone number. Next, they receive an information package. This package contains a study leaflet explaining the background of the study and instructions on when and how to perform the structured interview with the participant. An instruction card and checklist for this interview are also included. Then, they receive an instruction phone call from the research team four weeks after delivery. Finally, an email is sent to respective supervisors six weeks after delivery as a reminder for the phone-call. The supervisors are asked to perform the structured interview and to complete the accompanying checklist in the sixth week after delivery. They are requested to return the checklist by fax or email to the research team thereafter as soon as possible. If the checklist is not received at seven weeks after delivery, a reminder is sent by e-mail or made by telephone.

In the intervention group, supervisors are free to give their usual attention to employees who gave birth such as making home visits, calls or sending cards. If available, the policy of the company for pregnant employees or new mothers is allowed to be carried out during the study. For example, bringing a visit to the new mother with the "company's' bear" several weeks after childbirth.

#### Control group

Supervisors in the control group perform their common practice of acknowledging the arrival of their employees' newborn, for example, by making a home visit, calling or sending cards. If the participant is not able to RTW after maternity leave has ended, the standard sick-listing procedure is followed. If participants experience medical complaints during maternity leave, there is always the regular possibility of calling their supervisor or OHSP. Some OP's have reported before the start of the study that they routinely ask employees to contact them a few weeks before the end of maternity leave, in case of serious complaints during pregnancy.

### Blinding

Given the nature of the intervention, only the control group supervisors and participants are blinded to the intervention. The intervention group supervisors and participants cannot be blinded for the allocated treatment. Furthermore, the researcher and research assistant cannot be blinded for the allocated treatment. However, the data entry assistants are blinded for the allocated treatment. Data on sick leave and RTW are extracted from the routine databases of the company or the OHSP, thus, blinding does not play a role. The data will be blinded for the performance of the analysis. To guarantee blinding of the data for the analysis the participants, supervisors and companies are coded.

### Outcome assessment

All measurements, except sick leave data of OHSP or company, are performed by questionnaires. The baseline measurement consists of two parts: The first part (T0) when the employee makes herself known as participant; and the second part at 30 weeks pregnancy (T1). There is a one-year follow-up with assessments at six weeks (T2), 12 weeks (T3), 24 weeks (T4) and 52 weeks (T5) after the delivery date.

Units of health care use and productivity loss which are used to calculate costs are measured at six weeks (except work performance), 12 weeks (except work performance), 18 weeks, 24 weeks and 52 weeks after the delivery date. If a woman takes sick leave, has health care costs or has decreased work performance at 24 weeks, she receives a questionnaire on units of health care use and productivity loss again at 30 weeks, and if indicated by the same criteria also at 36, 42 and 48 weeks after the delivery date. Self-reported sick leave is assessed as part of the cost measurements, and objective sick leave data is obtained from the databases of the OHSP and, if available, from the databases of the company.

#### RCT primary outcome measure

The primary outcome measure for the RCT is work-status. Work-status is defined as sick leave and RTW. In this study, the following indicators are measured:

1) Time to partial and to full RTW, meaning number of calendar days between end of maternity leave and first day at work

2) Time to full RTW corrected for partial RTW

3) Partial and full RTW rate at 12, 24 and 52 weeks follow-up

4) Incidence of sick leave in the first year after delivery, following full RTW

5) Time to first recurrent sick leave in the first year after delivery, following full RTW

6) Total days of sick leave in the first year after delivery

7) Frequency of sick leave periods in the first year after delivery

#### RCT secondary outcome measures

In addition, data will be collected on the following items:

1. Costs are assessed from a societal perspective. Units of direct health care utilisation, both within and outside the health care sector, as well as productivity loss due to absenteeism and presenteeism are collected by cost diaries. Examples of direct health care units within the health care sector include consultations with the general practitioner, hospitalisations, and medications, and examples of direct health care units outside the health care sector include over-the-counter medication, informal care, and consultations with alternative health professionals. Costs will be calculated by multiplying the volume of resource use by cost prices, following the Dutch guidelines for economic evaluations in health care to estimate costs [15].

2. Health is measured with the RAND-36, a Dutch translation of the 36-Item Short Form Health Survey (SF-36) [16,17] and the EuroQol. The RAND-36 is a set of generic, coherent, and easily administered quality-of-life measures. It taps eight health concepts: physical functioning, bodily pain, role limitations due to physical health problems, role limitations due to personal or emotional problems, emotional well-being, social functioning, energy/fatigue and general health perceptions. It also includes a single item that provides an indication of perceived change in health. The Dutch version has a good reliability, good validity and is sensitive for changes [18]. Moreover, quality of life is also assessed with the EQ-5D also known as EuroQol (T2-T5) [19].

3. Satisfaction with the intervention is measured with the question, "Are you satisfied with the contact 6 weeks after delivery?", which can be answered on a six-point Likert scale. This question will be asked at 12 weeks after delivery.

4. Self-reported feelings of recovery from complaints, also called global perceived effect (GPE) is measured with the question, "To what extent have your complaints changed since 6 weeks after the delivery?", which can be answered on a seven-point Likert scale, with a range from "1" (completely recovered) to "7" (worse than ever). Again, this question will be asked at 12 weeks after delivery.

#### Cohort study outcome measures

In the cohort study, data will be collected on whether certain complaints develop or if RTW occurs among the participants.

1. The first outcome measure for the cohort is the presence of complaints of pregnancy-related pelvic girdle pain (PPGP). Two aspects on PPGP are measured: pain intensity and functional status. Pain intensity is measured with the same method as used in the Maastricht PPGP cohort study [20]. It is a self-report measure and contains questions whether the woman experiences pain at the lower back, buttocks, the symphysis pubis, groin or radiation into either or both legs on a eleven-point scale where zero is defined as "no pain" and 10 as "much pain". Functional status is measured with the Roland-Morris Disability-24 questionnaire (RDQ-24) [21]. The RDQ-24 is widely used in low back pain and pregnancy-related pelvic girdle pain research, and is tested by Riddle[22]. A validated Dutch version of the RDQ-24 is used in the study[23]. Both the test-retest reliability and construct validity of this questionnaire are considered good [22]. For the purpose of this study, the formulation of the items in the RDQ-24 was modified to read for 'my pelvic pain and/or low back pain' instead of 'my back pain'.

2. The second outcome measure for the cohort is fatigue, measured in two ways. Firstly, self-reported fatigue is rated on an eleven-point scale anchored by "0" for "no complaints" and "ten" for "many complaints". Secondly, fatigue is measured with the Checklist Individual Strength (CIS) questionnaire [24]. The CIS is a Dutch self-report questionnaire containing 20 items that are scored on a 7-point Likert scale [24,25]. The reference period of the scale is the past two weeks. Four aspects of fatigue are measured: subjective fatigue (somatic symptoms and general feelings of fatigue), reduction in motivation, reduction in concentration, and reduction in activity. The items do not refer to the work situation but are stated in general terms. The CIS is developed for clinical populations and has often been used in patients with chronic fatigue. Beurskens et al found that the CIS was able to discriminate between fatigued and non-fatigued employees in occupational groups [26].

3. The third outcome measure is depressive complaints. Three instruments are used to measure several aspects of depressive complaints. First, self-reported depressive feelings are rated on an eleven-point scale anchored by zero for "no complaints" and ten for "much complaints. Second, the Four Dimensional Symptom Questionnaire (4DSQ) is used under the assumption that mothers may experience distress as well as somatization. The 4DSQ is a Dutch self-rating questionnaire measuring four dimensions of common psychopathology: depression (severe anhedonia and depressive thoughts), anxiety (free floating anxiety, panic, and phobic anxiety), distress (non-specific symptoms of psychopathology), and somatization (a range of common physical symptoms)[27]. The 4DSQ is developed in general practice. Reliability of the 4DSQ scales is high and has good correlation. The factor structure of the 4DSQ has been confirmed[28]. Third, the Edinburgh Postnatal Depression Scale (EPDS) is used, because of its widely use in postnatal depression research. The EPDS is a 10-item self-rating depression scale[29]. The Dutch version of EPDS is found to be a self-rating scale with good psychometric characteristics [30]. In addition to depression, the instrument also measures anxiety [31].

#### Prognostic measures

Information is gathered on a number of variables that are considered to be prognostic factors for sick leave, RTW or for the development of complaints such as pregnancy-related pelvic girdle pain, fatigue and depression. Several categories of prognostic factors are distinguished: pregnancy-related, work-related, child-related, psychosocial-related and health-related prognostic factors. For each category a number of variables is measured with questionnaires. These variables are measured at different moments in time during the follow-up period. Some variables are measured with questions that have been developed specifically for the purpose of this study, while others are measured with validated measurement instruments. Table 1 shows an overview of the variables that are measured for each category, the measurement moments, and if applicable, the name of the validated instrument. Also, data are collected on possible confounders, such as age, education, marital status and ethnicity.

**Table 1:** Prognostic factors

	**T0**	**T1**	**T2**	**T3**	**T4**	**T5**
**Pregnancy-related factors:**						
Complaints and sick leave during pregnancy	x	x	x			
Previous pregnancies: birth dates, complaints and sick leave	x					
Fertility	x					
Delivery: duration, at home or in hospital, experience, complications and amount of rest after delivery			x			
Maternity and parental leave; length, satisfaction			x	x		x
Breastfeeding: length and experience at work with breastfeeding or expressing milk and facilities at work			x	x	x	x
**Work-related factors:**						
Work characteristics: function, number of working hours a week, working days, physical demands, extra work, travel time, shift work, adaptation of working hours or work tasks and work satisfaction	x			x		
Decision authority, skill discretion, psychological demands, social support of executive and colleges; Job Content Questionnaire [32,33]	x				x	
Total workload; hours a week spend on household, gardening, cycling, walking, biking, sport and odd jobs		x	x	x	x	x
Looking forward to returning to work			x	x		
Work performance; World Health Organization Health and Work Performance Questionnaire (HPQ) [34], also be measured at 18, 30, 36, 42, and 48 weeks after delivery (see outcome assessments, costs)					x	x
**Child-related factors:**						
Rang order child	x					
Birth weight child			x			
Health of child			x	x	x	x
Temperament of child; Childcare Stress Inventory [35,36]			x	x		
Childcare: childcare arrangement and changes in childcare during past year			x	x	x	x
**Psycho-Social factors:**						
Daily hassles; short version of the Everyday Problems Checklist (EPCL) [37,38]		x		x		
Social support of supervisor, colleagues, spouse, family and friends; Social Support List [39-41]		x	x		x	x
Norms about childcare and work; statements from a report of The Netherlands Interdisciplinary Demographic Institute NIDI [42]		x				x
Coping; Utrecht Coping List (UCL) [43,44]		x				
Locus of health control; Multidimensional Health Locus of Control List (MHLCL) [45].		x				
Life events		x	x	x	x	x
Difficulty of leaving child at home or day-care, when the mother is working			x	x	x	
**Health-related factors:**						
Body weight and body height	x		x	x	x	x
Smoking behaviour and alcohol intake	x					
Sleep quality		x	x	x	x	x
Co-existent chronic diseases		x				
(pregnancy-related) pelvic girdle pain (see outcome cohort study)		x	x	x	x	x
PPGP aspects: pain during rest, pain at beginning of movement, pain peak, diagnosis and treatment		x	x	x		
Fatigue (see outcome cohort study)		x	x	x	x	x
Depression (see outcome cohort study)		x	x	x	x	x
Pain catastrophizing; Pain Catastrophizing Scale (PCS) [46,47]		x				
Fear of movement; Tampa scale of Kinesiophopbia (TSK) [48]			x			

T0 = baseline, between 12 weeks and 30 weeks pregnancy, T1 = 30 weeks pregnancy, T2= 6 weeks postpartum (pp), T3 = 12 weeks pp, T4 = 24 weeks pp, T5 52 weeks pp

#### Process evaluation

One-third of the participants and supervisors in the intervention group receive a short questionnaire in order to evaluate the process of the intervention. Questions about their experience of the intervention are asked as well as which factors influenced according to them the effect of the intervention. One-third of the participants in the control group also receive a short questionnaire that asks about anticipated problems related to returning to work and whether these were discussed with their supervisor. Finally, at 12 weeks after delivery, all participants are asked if they had received any attention of their supervisor, whether their supervisor had talked about RTW and whether they were satisfied about this contact. They are also asked if they had received support of the OHSP and if they were satisfied about this support.

### Sample size

Van Beukering found that in 2002 about 30% of the women were taking sick leave after the maternity leave has ended[1]. In the Mom@Work study a decrease of 10% in sick leave at the end of the maternity leave to 20% (30–10 = 20%) is expected in the intervention group. To detect this 10% difference in sick leave, a minimum of 275 women is required per group. This difference can be detected with a power (1-β) of 80% at α= 0.05. A total of at least 550 women is needed for the entire study.

### Analysis

For the RCT, data will be analysed using statistical tests such as linear regression analysis and Cox regression analysis. The dependency of the observations will be taken into account by performing a multilevel analysis. The multilevel analysis will be performed with a four-level data structure where time, participants, supervisors and companies will be defined as levels 1, 2, 3 and 4, respectively. The analyses will be adjusted for baseline and prognostic dissimilarities, if necessary. Both an intention-to-treat analysis and a per-protocol analysis will be performed. For the cohort study, multilevel analysis will be performed with a three-level data structure where time will be defined as level 1; participants, level 2; and the companies, level 3.

The economic evaluation, which will be performed from a societal perspective, will consist of an analysis of differences in the total, direct and productivity loss costs between the intervention and control group, as well as a cost-effectiveness and cost-utility analysis of the intervention compared to common practice. In the cost-effectiveness analysis, sick leave will be the effect measure and the total costs will comprise all costs except those of productivity loss. Utilities will be based on the Euroqol questionnaire [19]. Quality Adjusted Life Years (QALY) will be calculated by multiplying the utility with the amount of time a patient spends in this particular health state. Incremental costs per QALY gained will be calculated applying both Dutch and UK tariffs. Bootstrapping methods will be used to estimate 95% confidence intervals for the incremental cost-effectiveness ratios and to derive cost-effectiveness planes and acceptability curves.

## Discussion

The Mom@Work study is designed to provide information about RTW of employees after giving birth. The effect of structural contact during maternity leave on sick leave will be evaluated, and factors that contribute to the development of complaints after childbirth will be assessed. By prospectively following a group of 550 pregnant workers until one year after delivery, much information will be gathered. A discussion of methodological issues of the Mom@Work study follows below.

In the intervention group, the supervisor contacts the participant six weeks after the delivery. An assumption was made that six weeks is a good moment to perform the intervention. In The Netherlands, most women have their last consult with their midwife, obstetrician or general practitioner at six weeks postpartum. It is assumed that, in general, women would be physically rehabilitated from delivery after 6 weeks. In The Netherlands, women have between 10–12 weeks of maternity leave after the delivery, before they have to start working again. When problems occur at six weeks after delivery, there is still sufficient time to solve those problems or to start prevention of long-term sick leave after maternity leave. Early identification of employees on sick leave who are at risk for long-term sick leave and work disability is important for starting early interventions. Therefore, it is crucial to seek early contact with those employees [8-11].

It may be argued that another time point than six weeks after delivery would be a better moment to intervene. Some women extend their leave by taking vacation, fulltime unpaid leave, or fulltime parental leave for a few days up to three months. At six weeks, postpartum it may be difficult to estimate the ability to resume work on an advanced date. Even when a woman does not extend her leave, it may be hard to estimate if one can truly start working again four to six weeks later, because during this period changes can occur to the mother or to the child, or one might feel healthy, but not healthy enough to work.

The supervisor is the person who contacts the participant in the intervention group. In our opinion, the supervisor is the most appropriate person to talk about RTW after maternity leave with their employees. The supervisor usually represents the employer in case the employee gets sick. Most companies have a policy that in case of sickness the supervisor keeps in contact with the employee. Although maternity leave is a special period, because the employee is not sick listed, employees are absent from work for at least 16 weeks.

The relationship between supervisor and employee may be important for the success of the intervention. It is possible that eligible employees refuse to participate, because of a poor relationship with their supervisor. In case employees and supervisors have a 'good' relationship they probably communicate better, than employees and supervisors who have a 'bad' relationship. This better communication may have the same effect as the structural contact in the intervention group, which may lead to a smaller difference between the intervention and control group. Therefore, all women will be asked if they had any conversation with their supervisor about RTW during maternity leave.

For practical reasons, we invited large companies with mostly female employees, and in particular, health care companies. This may have led to selection-bias and the results may probably not be generalisable to all (pregnant) working women. On the other hand, the results of the Mom@Work study will be most interesting for companies were a large number of women is working, and will also give an indication for problems and solutions for smaller companies or companies with a small number of female employees.

This study will provide important information about RTW of employees after giving birth. Results will give insight into the prognosis of sick leave and complaints after childbirth. Also, the role of the supervisor and the occupational physician in prevention of sick leave after childbirth will be clarified.

## Abbreviations

EPDS Edinburgh Postnatal Depression Scale

4DSQ Four Dimensional Symptom Questionnaire

HR Human Resource department

OP Occupational Physician

OHSP Occupational Health Service Provider

PPGP Pregnancy-related Pelvic Girdle Pain

QALY Quality Adjusted Life Years

RAND-36 36-Item Short Form Health Survey

RCT Randomised Controlled Trial

RDQ-24 Roland-Morris Disability Questionnaire

RTW Return-to-Work

CIS Checklist Individual Strength

## Competing interests

The author(s) declare that they have no competing interests.

## Authors' contributions

SGMS is performing the data collection and drafted the manuscript. MNMvP originated the idea for the study and is project-leader of the study. KU and MCdB are responsible for the cost-effectiveness data and analysis. SGMS, MNMvP, IJMH, DJB en WvM participated in the design of the study and research protocol. All authors read and corrected draft versions of the manuscripts and approved the final manuscript.

## Pre-publication history

The pre-publication history for this paper can be accessed here:


